# Major medical events in patients with acute coronary syndrome during helicopter emergency medical service operations

**DOI:** 10.1186/s12873-025-01308-7

**Published:** 2025-08-02

**Authors:** Julian Ganter, Hans-Jörg Busch, Alina Henis, Florian Reifferscheid, Jörg Braun, Sebastian Heinrich

**Affiliations:** 1 Department of Anesthesiology and Critical Care, Faculty of Medicine, Medical Center - University of Freiburg, University of Freiburg, Hugstetter Str. 55, 79106 Freiburg, Germany; 2 German Air Rescue Service Association DRF Luftrettung, Rita-Maiburg-Str. 2, 70794 Filderstadt, Germany; 3 Department of Emergency Medicine, Faculty of Medicine, Medical Center - University of Freiburg, University of Freiburg, Sir- Hans-A-Krebs-Str., 79106 Freiburg, Germany; 4 Department of Anesthesiology and Intensive Care Medicine, University Hospital Schleswig-Holstein, Campus Kiel, Kiel, Germany

**Keywords:** Emergency medicine, Cardiac arrest, Defibrillation, Risk stratification, Air rescue service

## Abstract

**Background:**

Acute coronary syndromes (ACS) are a leading cause of helicopter emergency medical services (HEMS) operations in Germany. Complications that arise during HEMS operations are challenging due to limited resources. However, the National Advisory Committee for Aeronautics (NACA) score and National Early Warning Score (NEWS) provide potential for risk stratification. Nevertheless, there is an absence of data concerning the incidence and risk of medical events (e.g. malignant arrhythmia, cardiac arrest, cardiogenic shock) in ACS patients during HEMS operations. The objective of this study is to evaluate the incidence of medical events and to assess risk stratification using scoring systems.

**Methods:**

This retrospective observational cohort study analyzed prehospital records from 38,473 HEMS operations with “ACS” coding conducted between 2012 and 2024 in Germany. Routine data were systematically recorded using a standardized digital form that captured patient demographics, clinical presentation, and medical interventions. Major medical events (MME) were defined using surrogate markers, including defibrillation, resuscitation, airway management, ventilation, and new ST-elevation myocardial infarction (STEMI) findings. Scores (NACA, NEWS, and a combined MME-score) were calculated, with the MME-score integrating NACA and NEWS. Ethical approval was obtained from the Albert-Ludwigs-University Freiburg Ethics Committee (No: 24-1082-S1, 25 April 2024).

**Results:**

MME occurred in 8.8% of the 38,473 HEMS operations. They occurred more frequently during secondary missions (interfacility transports) (11.8%) than primary missions (6.7%), and at night (15.3%) than during the day (8.2%) (both *p* < 0.001). The NACA, NEWS, and MME-scores were significantly higher in cases with medical events (*p* < 0.001). The risk stratification exhibited areas under the curve in the receiver operating characteristics (ROC) curve, with values of 0.831 for NACA, 0.866 for NEWS, and 0.895 for the MME-score.

**Conclusion:**

The incidence of MMEs is subject to variation depending on the operational context. Established scoring systems such as the NACA and NEWS are available for the purpose of risk stratification of medical events in patients with ACS during air rescue operations. The combination of these scores may indicate potential for improved risk stratification.

**Supplementary Information:**

The online version contains supplementary material available at 10.1186/s12873-025-01308-7.

## Background

Acute coronary syndromes (ACS) are among the most common reasons for emergency medical services (EMS) activations. The DRF Luftrettung, a leading air rescue organization in Germany, conducts about 35,000 operations yearly, with roughly 20% related to cardiac conditions [1]. Due to the risk of complications that may necessitate medical intervention during air transport, these patients are typically transferred to the nearest hospital. Primary missions enable the rapid access of emergency physicians to patients in urban and rural environments. Secondary missions (interfacility transports) involve transporting patients from a general care hospital to another where they can receive specialized treatment [2, 3]. However, medical events (events requiring acute intervention, e.g. malignant arrhythmia, cardiac arrest, cardiogenic shock) during air transport requiring urgent responses can arise in any type of mission and should be anticipated and managed promptly. Analyzing the risk of complications that may require medical intervention during the flight allows for better preparation and informed consideration of additional options for action. In light of resource constraints and the decreasing availability of physician-based emergency medical services on the one hand and the establishment of tele-emergency medical systems along with the enhanced qualifications of emergency medical personnel on the other, predicting the risk of events that require intervention during transport can serve as a valid decision-making aid for choosing the appropriate transport modality. Effective in-flight care is challenging even for highly qualified and specialized teams [4, 5].

However, the actual occurrence of medical events requiring intervention has not been previously investigated with large case numbers. Currently, no comprehensive data set for risk stratification regarding predicting in-flight complications in patients suffering from ACS is available. The National Advisory Committee for Aeronautics (NACA) score is often used within the EMS system as a tool for risk stratification [6, 7]. However, accurate data on ACS patients involved in air rescue operations remains deficient. There are established in-hospital early warning scores, like the National Early Warning Score (NEWS), which can be used to determine risk stratification based on easily accessible basic parameters [8].

The objective of this study was to evaluate the incidence of events requiring acute intervention and to assess risk stratification using scoring systems in patients with ACS during helicopter emergency medical service (HEMS) operations.

## Methods

### Study design and data processing

This retrospective observational cohort study analyzed the routine documentation of HEMS operations over a 12-year period (2012–2024) from all air rescue stations in Germany operated by the DRF Luftrettung (DRF, Filderstadt Germany) with the coding “ACS patient”. Each helicopter crew comprises at least a pilot, a HEMS Technical Crew Member (HEMS-TC), and an emergency physician. The nighttime operations were conducted with an additional pilot. As a crucial component of the public emergency medical service (EMS), the helicopters are dispatched by local emergency dispatch centers alongside ambulances for primary operations. Secondary missions for interfacility transports are coordinated by cross-regional coordination centers and carried out independently of other EMS resources.

The operational data of the helicopters is systematically recorded in a dedicated database. From 2012 to August 2022, each operation was documented using a standardized digital form (HEMSDER Database, Convexis, Germany). After August 2022, the documentation was performed using the NIDApad System (medDV GmbH, Fernwald, Germany). Both systems capture a nearly identical data set that includes patient demographic information, clinical presentation and operational data, including relevant timestamps and specifics of medical interventions related to diagnostics and therapeutic measures.

### Definitions

Since events during operations are not documented with sufficient accuracy and quality in practice, surrogate markers have been defined to represent “major medical events” (MME) and have subsequently been used as a composite outcome. Unlike the events themselves, these surrogate markers are documented with high reliability. The following MME were defined along with their corresponding surrogate markers:

- Malignant arrhythmia (Defibrillation, CPR, Ventilation, airway management).
- Cardiac arrest (Defibrillation, CPR, Ventilation, airway management).
- Heart failure / cardiogenic shock (CPR, Ventilation, airway management).
- New STEMI on ECG (STEMI not initial but documented at handover).

For this study, the NACA score was used, determined by the emergency physician based on the patient’s most severe clinical state throughout the entire mission. The original NEWS score was applied, which uses SpO₂ Scale 1 as later defined in NEWS2 [9]. For the assessment of consciousness, the Glasgow Coma Scale (GCS) was categorized, with scores of 13–15 classified as “A” (Alert) and scores of 3–12 classified as “VPU” (Verbal, Pain, or Unresponsive) [10]. The NEWS2 score was calculated whenever at least one required parameter was available. The addition of NACA and NEWS created the MME-score and can therefore assume natural numbers between 0 and 27. Nighttime was documented when the core of the operation was accomplished after sunset or before sunrise.

### Statistical analysis and ethical approval

Continuous variables were described and compared using the mean and standard deviation. Nominal variables were analyzed using the Pearson Chi-Square Test. The Pearson correlation was applied for two-sided testing, and effect sizes were evaluated with Cohen’s d for independent samples. A t-test was conducted for independent samples. A receiver operating characteristic (ROC) analysis was performed to assess the specificity and sensitivity of the selected scores [11]. All tests were considered significant for p values < 0.05. Analysis was conducted using IBM SPSS Statistics 29 (Armonk, NY, USA).

The study was approved by the Ethics Committee of the Albert-Ludwigs-University Freiburg (No: 24-1082-S1, 25 April 2024) and registered with the German Register of Clinical Studies (No: DRKS00033536, 1 February 2024).

## Results

### Characteristics

**Fig. 1:**
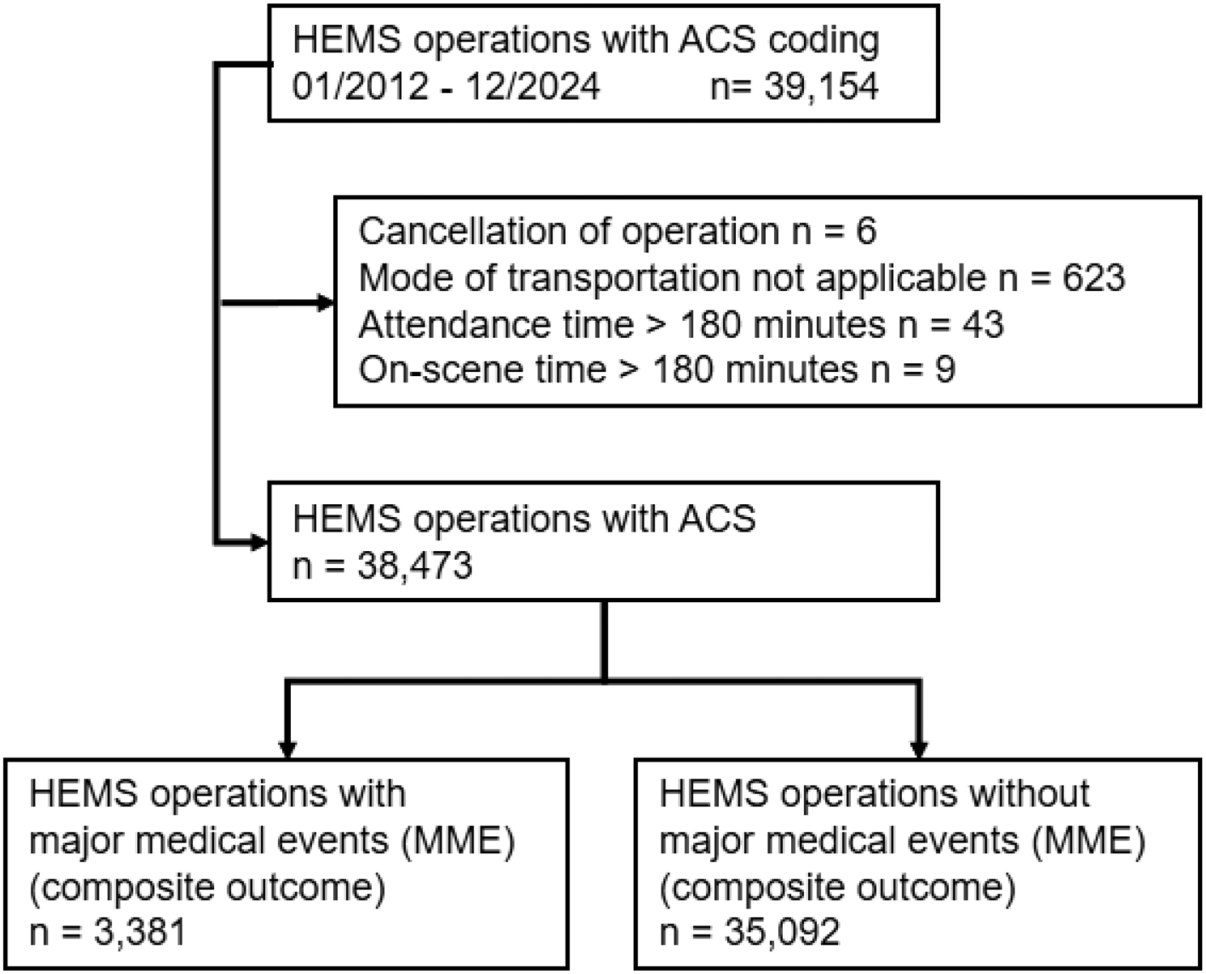
CONSORT Flow diagram

The study included 38,473 HEMS missions with “ACS” coding from January 1, 2012, to December 31, 2024 (see Fig. 1). MME, including defibrillation, CPR, airway management, ventilation, and new STEMI ECG, occurred in 8.8% (3,381/38,473). MME occurred in 6.7% (1,514) during primary operations and in 11.8% (1,867/15,882) during secondary operations (*p* < 0.001). In 8.2% (2,880/35,157) of the daytime operations and in 15.3% (499/3,252) (*p* < 0.001) of the nighttime operations at least one MME was recorded. All characteristics are shown in Table 1.

**Table 1:** Characteristics of all helicopter emergency medical service (HEMS) operations of patients with acute coronary syndrome (ACS) with subdivision into operations with/without major medical events (MME)

	HEMS missions with MME	HEMS missions without MME	Overall
Missions (n, %)	3.381 (8.8%)	35.092 (91.2%)	38.473
Patients			
Age (in years, mean, n)	66.3 ± 12.4 (3,381)	67.8 ± 13.4 (35,088)	67.6 ± 13.28 (38,469)
Male gender (n)	2,457	23,437	25.894 (67.3%)
Female gender (n)	843	10,884	11.727 (30.5%)
Unknown gender (n)	81	771	852 (2.2%)
Process			
Primary operations (%, n)	6.7% (1,514)	93.3% (21,077)	22.591 (58.7%)
Secondary operations (%, n)	11.8% (1,867)	88.2% (14,015)	15.882 (41.3%)
Day operations (%, n)	8.2% (2,880)	91.8% (32,277)	35.157 (91.4%)
Night operations (%, n)	15.3% (499)	84.7% (2,753)	3.252 (8.5%)
Not determinable (%, n)	3.1% (2)	96.9% (62)	64 (0.2%)
Mode of transportation			
with helicopter (%, n)	10.7% (2.404)	89.3% (20.063)	22.467 (58.4%)
HEMS physician with ambulance (%, n)	6.4% (873)	93.6% (12.692)	13.565 (35.3%)
Handover to other means of rescue (%, n)	2.2% (49)	97.8% (2.226)	2.275 (5.9%)
On-Site handover (%, n)	33.1% (55)	66.9% (111)	166 (0.4%)
Major Medical Events			
Defibrillation (%, n)	465	0	465 (1.2%)
CPR (%, n)	927	0	927 (2.4%)
Intubation (%, n)	2,441	0	2,441 (6.3%)
Ventilation (%, n)	2,904	0	2,904 (7.5%)
New STEMI finding (ECG) (%, n)	177	0	177 (0.5%)

SD = standard deviation, ECG = Electrocardiogram, STEMI = ST-Elevation Myocardial Infarction, CPR = cardiopulmonary resuscitation)

### NACA, NEWS and MME-score

**Fig. 2:**
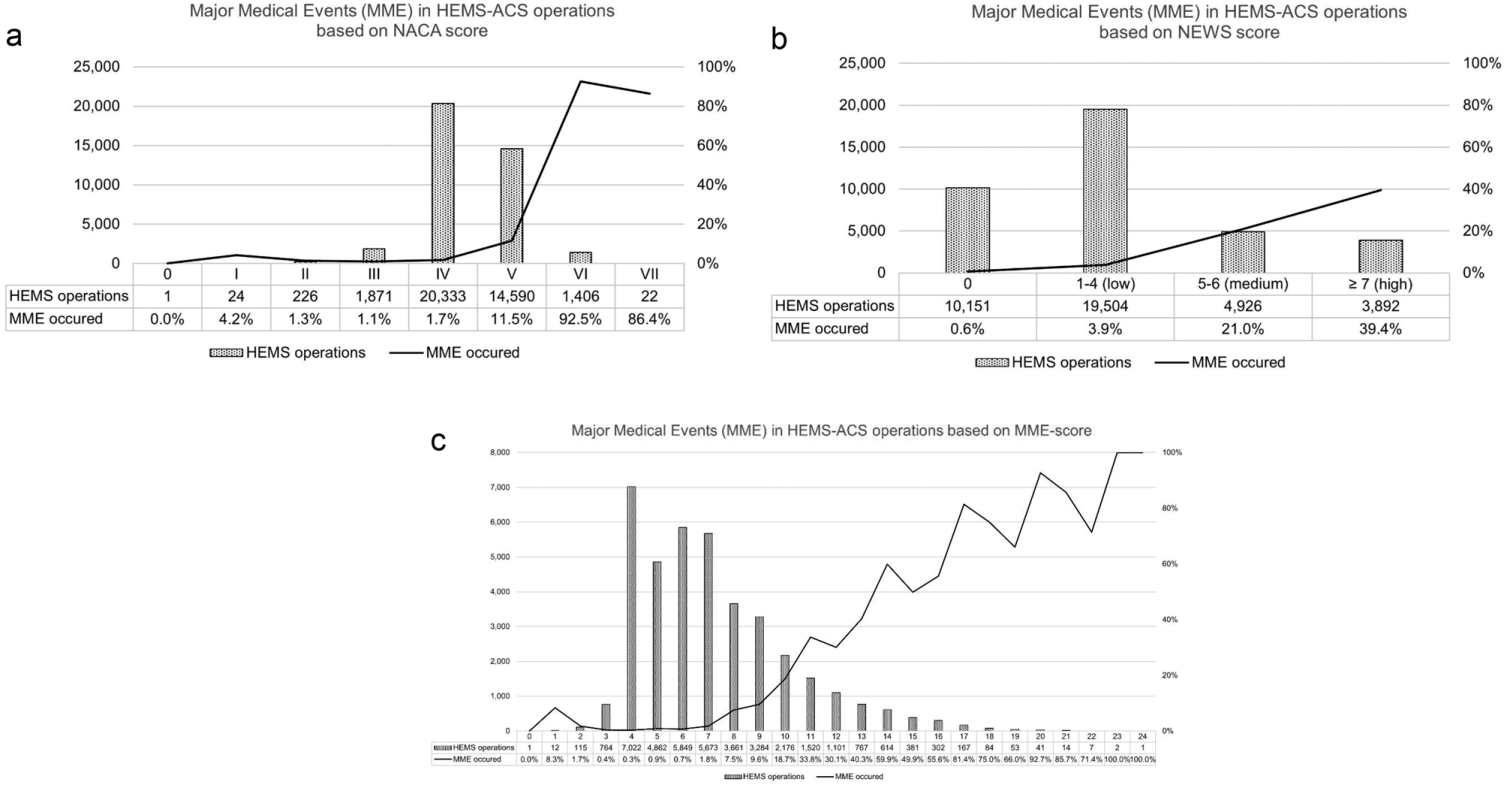
**a** Proportion of Major Medical Events (MME) in HEMS-ACS operations based on NACA score. **b** Proportion of Major Medical Events (MME) in HEMS-ACS operations based on NEWS score. **c** Proportion of Major Medical Events (MME) in HEMS-ACS operations based on MME-score (single summative score of NACA and NEWS score)

The NACA score was a mean of 4.39 ± 0.68 overall. NACA scores with and without MME were 5.28 ± 0.67 and 4.31 ± 0.61 (*p* < 0.001) (Fig. 2a). The mean NEWS score was 2.78 ± 2.69 overall. The NEWS scores, with and without MME, were 6.56 ± 3.01 and 2.42 ± 2.36 (*p* < 0.001), respectively (Fig. 2b). The MME-score shows a mean of 7.18 ± 2.99 overall. The MME-scores with and without MME were 11.83 ± 3.23 and 6.72 ± 2.55 (*p* < 0.001) (Fig. 2c). The detailed results for each value of the NACA, NEWS, and MME-scores are shown in Table 2.

**Table 2:** NACA, NEWS and MME-score of all helicopter emergency medical service (HEMS) operations of patients with acute coronary syndrome (ACS) with subdivision into operations with/without major medical events (MME)

	HEMS missions with MME	HEMS missions without MME	Overall
Operations (n, %)	3,381 (8.8%)	35,092 (91.2%)	38,473
NACA Score			
NACA (mean, SD)	5.28 ± 0.67	4.31 ± 0.61	4.39 ± 0.68
NACA 0 (%, n)	0.0% (0)	100.0% (1)	1 (0.0%)
NACA I (%, n)	4.2% (1)	95.8% (23)	24 (0.1%)
NACA II (%, n)	1.3% (3)	98.7% (223)	226 (0.6%)
NACA III (%, n)	1.1% (21)	98.9% (1,850)	1,871 (4.9%)
NACA IV (%, n)	1.7% (352)	98.3% (19,981)	20,333 (52.9%)
NACA V (%, n)	11.5% (1,684)	88.5% (12,906)	14,590 (37.9%)
NACA VI (%, n)	92.5% (1,301)	7.5% (105)	1,406 (3.7%)
NACA VII (%, n)	86.4% (19)	13.6% (3)	22 (0.1%)
NEWS Score			
NEWS (mean, SD)	6.56 ± 3.01	2.42 ± 2.36	2.78 ± 2.69
NEWS 0 (%, n)	0.6% (60)	99.4% (10,091)	10,151 (26.4%)
NEWS 1–4 (low) (%, n)	3.9% (751)	96.1% (18,753)	19,504 (50.7%)
NEWS 5–6 (medium) (%, n)	21.0% (1,035)	79.0% (3,891)	4,926 (12.8%)
NEWS ≥ 7 (high) (%, n)	39.4% (1,535)	60.6% (2,357)	3,892 (10.1%)
MME-score			
MME (mean, SD)	11.83 ± 3.23	6.72 ± 2.55	7.18 ± 2.99
MME 0 (%, n)	0.0% (0)	100.0% (1)	1 (0.0%)
MME 1 (%, n)	8.3% (1)	91.7% (11)	12 (0.0%)
MME 2 (%, n)	1.7% (2)	98.3% (113)	115 (0.3%)
MME 3 (%, n)	0.4% (3)	99.6% (761)	764 (2.0%)
MME 4 (%, n)	0.3% (21)	99.7% (7,001)	7,022 (18.3%)
MME 5 (%, n)	0.9% (43)	99.1% (4,819)	4,862 (12.6%)
MME 6 (%, n)	0.7% (43)	99.3% (5,806)	5,849 (15.2%)
MME 7 (%, n)	1.8% (100)	98.2% (5,573)	5,673 (14.7%)
MME 8 (%, n)	7.5% (275)	92.5% (3,386)	3,661 (9.5%)
MME 9 (%, n)	9.6% (315)	90.4% (2,969)	3,284 (8.5%)
MME 10 (%, n)	18.7% (407)	81.3% (1,769)	2,176 (5.7%)
MME 11 (%, n)	33.8% (513)	66.2% (1,007)	1,520 (4.0%)
MME 12 (%, n)	30.1% (331)	69.9% (770)	1,101 (2.9%)
MME 13 (%, n)	40.3% (309)	59.7% (458)	767 (2.0%)
MME 14 (%, n)	59.9% (368)	40.1% (246)	614 (1.6%)
MME 15 (%, n)	49.9% (190)	50.1 (191)	381 (1.0%)
MME 16 (%, n)	55.6% (168)	44.4% (134)	302 (0.8%)
MME 17 (%, n)	81.4% (136)	18.6% (31)	167 (0.4%)
MME 18 (%, n)	75.0% (63)	25.0% (21)	84 (0.2%)
MME 19 (%, n)	66.0% (35)	34.0% (18)	53 (0.1%)
MME 20 (%, n)	92.7% (38)	7.3% (3)	41 (0.0%)
MME 21 (%, n)	85.7% (12)	14.3% (2)	14 (0.0%)
MME 22 (%, n)	71.4% (5)	28.6% (2)	7 (0.0%)
MME 23 (%, n)	100.0% (2)	0.0% (0)	2 (0.0%)
MME 24 (%, n)	0.0% (1)	0.0% (0)	1 (0.0%)
MME 25 (%, n)	− (0)	− (0)	0.0% (0)
MME 26 (%, n)	− (0)	− (0)	0.0% (0)
MME 27 (%, n)	− (0)	− (0)	0.0% (0)

SD = standard deviation, NACA = National Advisory Committee for Aeronautics, NEWS = National Early Warning Score

**Fig. 3:**
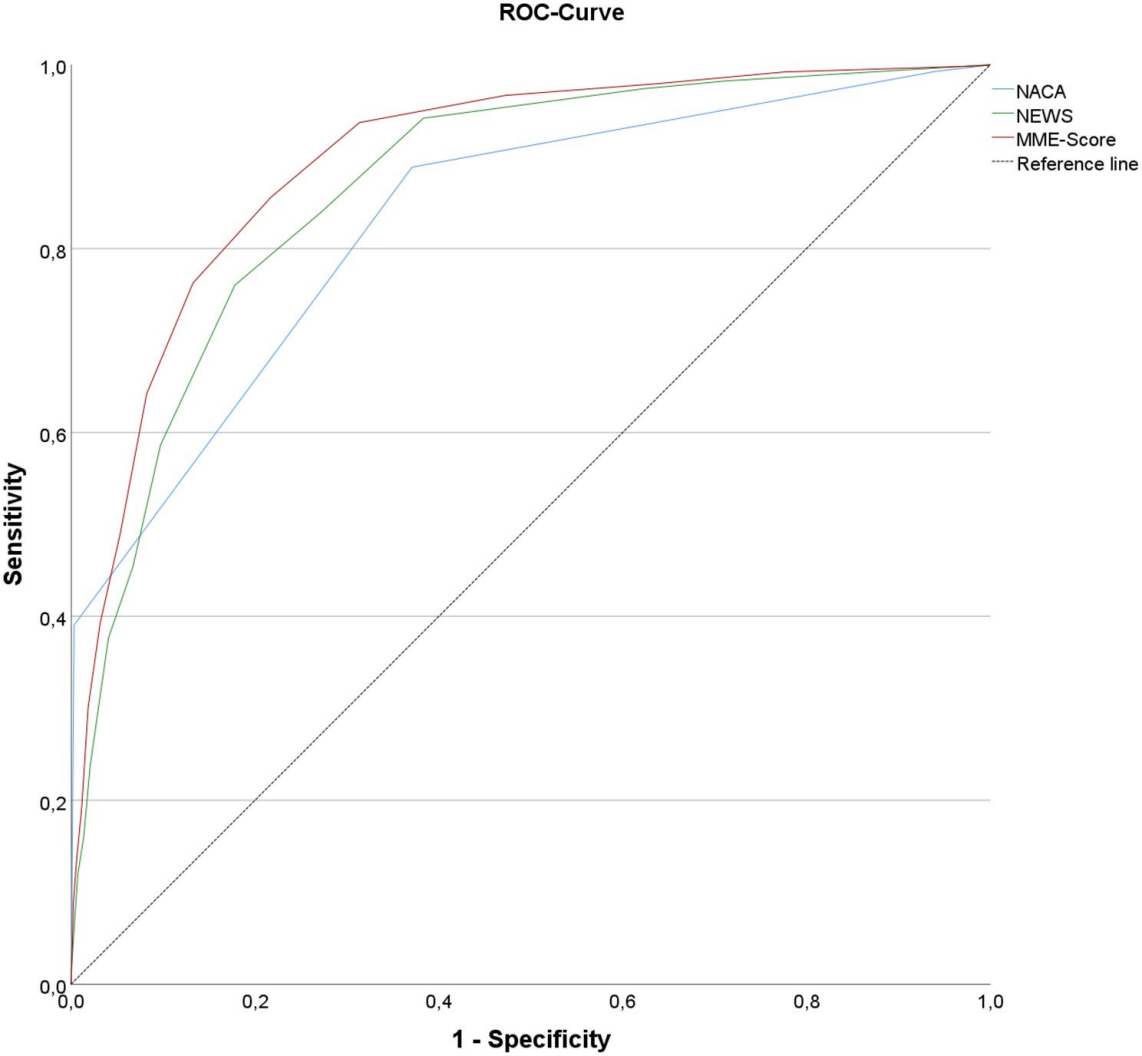
ROC-Curve for NACA, NEWS and MME-score

The area under the curve (AUC) of the receiver operating characteristic (ROC) curve utilized in the analysis of MME occurrence yielded the following results: 0.831 (95% CI: 0.823–0.839) for the NACA score, 0.866 (95% CI: 0.860–0.872) for the NEWS score, and 0.895 (95% CI: 0.890–0.900) for the combined score (Fig. 3).

## Discussion

According to the documented NACA score, this study shows that patients with ACS for whom HEMS is dispatched are typically in critical condition. However, the occurrence of specifically defined MME within this patient group seems relatively low. Early risk stratification is crucial and can be effectively achieved using the NACA and NEWS scores. While more specialized scoring systems such as the GRACE, HEART or TIMI scores are designed to assess risk in patients with acute coronary syndrome, they are primarily suited for use in emergency units or intensive care settings and help to guide clinical decision-making [12].

In general, the incidence of MME in primary operations is rather low at around 7% of all operations. In contrast, the higher incidence of around 12% observed in secondary interventions may reflect the fact that these patients had already been classified as critically ill in a clinical setting and required urgent transfer to specialized medical facilities [2, 3, 13]. A comparison between daytime and nighttime operations further revealed a higher rate of MME during nighttime (15% vs. 8%). The overall complexity of conducting operations at night is considerably greater than during daylight hours [14–16]. Only a limited number of air rescue centers in Germany operate on a 24/7 basis. Consequently, it can be assumed that operations during nighttime are conducted when absolutely essential, implying that the most critically ill patients are transported by HEMS during these hours [17]. Night operations principally demand greater resources, as navigation must be assisted either by a second pilot or the HEMS-TC. Additionally, restricted visibility presents an increased risk to the crew, necessitating supplementary measures such as the illumination of landing sites by fire services.

The NACA score is widely applied in prehospital settings to rapidly assess the severity of illness or injury and guide operational decisions. It also serves as a tool for quality assurance and data reporting [18, 19]. More recent studies have also demonstrated an association with mortality [20] or noted life-threatening cardiac arrhythmias in patients with NACA greater than IV [6]. However, the NACA score relies on a subjective clinical impression and thus cannot be considered an objectifiable score, even though validation using measurable parameters indicates a correlation [18]. Nevertheless, modified NACA scores are employed in certain domains with the objective of attaining enhanced precision by integrating quantifiable data [21]. Recently, there have been considerations to assess the NACA score not only initially but also at handover, utilizing bitemporal scoring. The goal of reassessing and documenting the score twice is to provide insights into the patient’s progression and the effectiveness of prehospital treatment [22]. However, the most unfavorable NACA score remains crucial for predictive purposes and was therefore employed in this study.

To address the issue of score objectification, this study should include: (1) standard parameters that are easy to collect and (2) existing standard parameters in the analysis. The NEWS score meets the outlined requirements, even though its application and validation are primarily based on in-hospital use [8, 15, 16]. In the past, NEWS score has performed moderately accurate in the prehospital setting [17].

Based on the available results, the NEWS score reflects good to very good performance within this study cohort. Combining the NACA and NEWS scores into a single summative score was not only practical but, although not yet validated, may indicate a potential for improved risk stratification, as reflected by the AUC (0.895). This value indicates a high level of accuracy in distinguishing between patients with and without the risk of MME. The MME-score provides substantial support, especially in identifying patients whose condition falls between mild illness (NACA I-II) and critical severity (NACA V-VII). The data show that this intermediate patient group, marked by a NACA score of IV, makes up the majority of HEMS ACS interventions, representing 52.9% of all operations.

The integration of the MME-score into an existing documentation system is quite simple. The parameters of the NEWS score align with standard vital sign measurements, and the NACA score is already routinely documented. By incorporating this system into the documentation software, the MME-score (sum of the NACA and NEWS scores) could be automatically calculated and recorded on a regular basis. This would provide the HEMS crew with an early indication of potential MME as soon as the initial vital parameters are logged. Furthermore, the MME-score introduced in this study could be continuously reassessed and validated through systematic and ongoing documentation in future applications. While the integration of the MME-score offers clear operational benefits, it is also important to consider that reliance on a scoring system may require adjustments in existing workflows and additional training. Overreliance could potentially limit individualized patient care if the score is used without sufficient clinical context. The routine documentation of the MME score could also assist in deciding whether the HEMS should support the pilot in the front or the emergency physician in the cabin (medication preparation, ventilation, CPR equipment).

## Limitations

This study is subject to several important limitations. Owing to its retrospective design and reliance on routinely collected documentation, key potential confounders (such as interfacility transfer distances, cardiovascular event subtypes, crew experience, weather conditions, and equipment availability) could not be accounted for. The NACA score, based on the individual clinical judgment of the attending emergency physician, may have been influenced by variations in professional background and experience, which were not captured in the dataset. While all participating HEMS stations are staffed by emergency physicians with standardized and comparable qualifications, this differs from many international EMS and HEMS systems where physicians are not routinely involved in prehospital care. Furthermore, as this study is limited to the prehospital setting, no conclusions can be drawn regarding longer-term patient outcomes. Prospective studies are warranted to further evaluate the utility of the MME score, particularly following the systematic integration of the NEWS score into routine documentation.

## Conclusion

This study indicates that relevant MME occur in a number of HEMS missions, with increased rates being observed during secondary missions and nighttime operations. The NACA and NEWS scores can be readily calculated using routine documentation, facilitating seamless integration into existing systems. The combination of these scores may offer a pragmatic tool to improve risk stratification accuracy. Exploration of the potential of these simple scoring systems to improve risk assessment is essential for determining their effectiveness in resource allocation, supporting clinical decision-making in the prehospital care, and ultimately enhancing patient safety and care.

## Electronic supplementary material

Below is the link to the electronic supplementary material.

Supplementary Material 1

## Data Availability

Reasonable requests to provide raw data material will be assessed by the review board of the scientific working group of German Air Rescue Service Association.

## Abbreviations

ACS: Acute Coronary Syndrome
CPR: Cardio Pulmonary Resuscitation
ECG: Electrocardiogram
EMS: Emergency Medical Service
HEMS: Helicopter Emergency Medical Service
HEMS-TC: HEMS Technical Crewmember
MME: Major Medical Event
NACA: National Advisory Committee for Aeronautics (Score)
NEWS: National Early Warning (Score)
STEMI: ST-Elevation Myocardial Infarction
