# Supplementary material for: Major medical events in patients with acute coronary syndrome during helicopter emergency medical service operations
Source: BMC Emerg Med. 2025 Aug 2;25:145. doi: 10.1186/s12873-025-01308-7 (PMC12318427; doi:10.1186/s12873-025-01308-7)
Supplement: Supplementary file 1 — Supplementary Material 1 [file 12873_2025_1308_MOESM1_ESM.docx]

**Supplement**

| **Category** | **Description** |
| --- | --- |
| **NACA 0** | No injury or disease.  This category is often deleted or replaced by NACA I. |
| **NACA I** | Minor disturbance. No medical intervention is required. |
| **NACA II** | Slight to moderate disturbance. Outpatient medical investigation, but usually no emergency medical measures necessary. |
| **NACA III** | Moderate to severe but not life-threatening disorder. Stationary treatment required, often emergency medical measures on the site |
| **NACA IV** | Serious incident where rapid development into a life-threatening condition cannot be excluded. In the majority of cases, emergency medical care is required |
| **NACA V** | Acute danger |
| **NACA VI** | Respiratory and/or cardiac arrest |
| **NACA VII** | Death |

**Supplement** NACA-Score

| **Observation** | **3** | **2** | **1** | **0** | **1** | **2** | **3** |
| --- | --- | --- | --- | --- | --- | --- | --- |
| **Respiratory rate (bpm)** | ≤ 8 |  | 9–11 | 12–20 |  | 21–24 | ≥ 25 |
| **Oxygen saturation (SpO2%)** | ≤ 91 | 92–93 | 94–95 | ≥ 96 |  |  |  |
| **Any Supplemental Oxygen** |  | Yes |  | No |  |  |  |
| **Temperature (°C)** | ≤ 35.0 |  | 35.1–36.0 | 36.1–38.0 | 38.1–39.0 | ≥ 39.1 |  |
| **Systolic blood pressure (mmHg)** | ≤ 90 | 91–100 | 101–110 | 111–219 |  |  | ≥ 220 |
| **Heart rate (bpm)** | ≤ 40 |  | 41–50 | 51–90 | 91–110 | 111–130 | ≥ 131 |
| **Level of consciousness** |  |  |  | Alert (A) |  |  | Verbal (V)  Pain (P)  Unresponsive (U) |

**Supplement** NEWS Score
